# P-values – a chronic conundrum

**DOI:** 10.1186/s12874-020-01051-6

**Published:** 2020-06-24

**Authors:** Jian Gao

**Affiliations:** Department of Veterans Affairs, Office of Productivity, Efficiency and Staffing (OPES, RAPID), Albany, USA

**Keywords:** *P*-values, Type I error, Significance testing, Hypothesis testing, Research reproducibility, Calibrated *P*-values

## Abstract

**Background:**

In medical research and practice, the *p*-value is arguably the most often used statistic and yet it is widely misconstrued as the probability of the type I error, which comes with serious consequences. This misunderstanding can greatly affect the reproducibility in research, treatment selection in medical practice, and model specification in empirical analyses. By using plain language and concrete examples, this paper is intended to elucidate the *p*-value confusion from its root, to explicate the difference between significance and hypothesis testing, to illuminate the consequences of the confusion, and to present a viable alternative to the conventional *p*-value.

**Main text:**

The confusion with *p*-values has plagued the research community and medical practitioners for decades. However, efforts to clarify it have been largely futile, in part, because intuitive yet mathematically rigorous educational materials are scarce. Additionally, the lack of a practical alternative to the *p*-value for guarding against randomness also plays a role. The *p*-value confusion is rooted in the misconception of significance and hypothesis testing. Most, including many statisticians, are unaware that *p*-values and significance testing formed by Fisher are incomparable to the hypothesis testing paradigm created by Neyman and Pearson. And most otherwise great statistics textbooks tend to cobble the two paradigms together and make no effort to elucidate the subtle but fundamental differences between them. The *p*-value is a practical tool gauging the “strength of evidence” against the null hypothesis. It informs investigators that a *p*-value of 0.001, for example, is stronger than 0.05. However, *p*-values produced in significance testing are not the probabilities of type I errors as commonly misconceived. For a *p*-value of 0.05, the chance a treatment does not work is not 5%; rather, it is at least 28.9%.

**Conclusions:**

A long-overdue effort to understand *p*-values correctly is much needed. However, in medical research and practice, just banning significance testing and accepting uncertainty are not enough. Researchers, clinicians, and patients alike need to know the probability a treatment will or will not work. Thus, the calibrated *p*-values (the probability that a treatment does not work) should be reported in research papers.

## Background

Without any exaggeration, humankind’s wellbeing is profoundly affected by *p*-values: Health depends on prevention and intervention, ascertaining their efficacies relies on research, and research findings hinge on *p*-values. The p-value is a sine qua non for deciding if a research finding is real or by chance, a treatment is effective or even harmful, a paper will be accepted or rejected, a grant will be funded or declined, or if a drug will be approved or denied by U.S. Food & Drug Administration (FDA).

Yet, the misconception of *p*-values is pervasive and virtually universal. “The *P* value is probably the most ubiquitous and at the same time, misunderstood, misinterpreted, and occasionally miscalculated index in all of biomedical research [1].” Even “among statisticians there is a near ubiquitous misinterpretation of *p* values as frequentist error probabilities [2].”

The extent of the *p*-value confusion is well illustrated by a survey of medical residents published in the *Journal of the American Medical Association* (*JAMA)*. In the article, 88% of the residents expressed fair to complete confidence in understanding *p*-values, but 100% of them had the p-value interpretation wrong [1, 3]. Make no mistake, they are the future experts and leaders in clinical research that will affect public health policies, treatment options, and ultimately people’s health.

The survey published in *JAMA* used multiple-choice format with four potential answers for a correct interpretation of *p* > 0.05 [3]:

- *The chances are greater than 1 in 20 that a difference would be found again if the study were repeated.*
- *The probability is less than 1 in 20 that a difference this large could occur by chance alone.*
- *The probability is greater than 1 in 20 that a difference this large could occur by chance alone.*
- *The chance is 95% that the study is correct.*

How could it be possible that 100% of the residents selected incorrect answers when one of the possible choices was supposed to be correct? As reported in the paper [3], 58.8% of the residents selected choice *c* which was designated by the authors as the correct answer. The irony is that choice *c* is not correct either. In fact, none of the four choices are correct. So, not only were the residents who picked choice *c* wrong but also the authors as well. Keep in mind, the paper was peer-reviewed and published by one of the most prestigious medical journals in the world.

This is no coincidence -- most otherwise great statistics textbooks make no effort or fail to clarify the massive confusion about *p*-values, and even provide outright wrong interpretations. The confusion is near-universal among medical researchers and clinicians [4–6].

Unfortunately, the misunderstanding of *p*-values is not inconsequential. For a p-value of 0.05, the chance a treatment doesn’t work is not 5%; rather, it is at least 28.9% [7].

After decades of misunderstanding and inaction, the pendulum of *p*-values finally started to swing in 2014 when the American Statistical Association (ASA) was taunted by two pairs of questions and answers on its discussion forum:

- Q: Why do so many colleges and grad schools teach *p* = 0.05?
- A: Because that’s still what the scientific community and journal editors use.
- Q: Why do so many people still use *p* = 0.05?
- A: Because that’s what they were taught in college or grad school.

The questions and answers, posted by George Cobb, Professor Emeritus of Mathematics & Statistics from Mount Holyoke College, spurred the ASA Board into action. In 2015, for the first time, the ASA board decided to take on the challenge of developing a policy statement on *p*-values, much like the American Heart Association (AHA) policy statement on dietary fat and heart disease. After months of preparation, in October 2015, a group of 20 experts gathered at the ASA Office in Alexandria, Virginia and laid out the roadmap during a two-day meeting. Over the next three months, multiple drafts of the *p*-value statement were produced. On January 29, 2016, the ASA Executive Committee approved the *p*-value statement with six principles listed on what *p*-values are or are not [8].

Although the statement hardly made any ripples in medical journals, it grabbed many statisticians’ attention and ignited a rebellion against *p*-values among some scientists. In March 2019, *Nature* published a comment with over 800 signatories calling for an end of significance testing with *p* < 0.05 [9]. At the same time, the *American Statistician* that carried the ASA’s *p*-value statement published a special issue with 43 articles exploring ways to report results without statistical significance testing. Unfortunately, no consensus was reached for a better alternative in gauging the reliability of studies, and the authors even disagreed on whether the *p*-value should continue to be used or abandoned. The only agreement reached was the abolishment of significance testing as summarized in the special issue’s editorial: “statistically significant” – don’t say it and don’t use it [10].

So, for researchers, practitioners, and journals in the medical field, what will replace significance testing? And what is significance testing anyway? Is it different from hypothesis testing? Should *p*-values be banned too? If not, how should p-values be used and interpreted? In healthcare or medicine, we must accept uncertainty as the editorial of the special issue urged, but do we need to know how likely a given treatment will work or not?

To answer these questions, we must get to the bottom of the misconception and confusion, and we must identify a practical alternative(s). However, despite numerous publications on this topic, few studies aimed for these goals are understandable to non-statisticians and retain mathematical rigor at the same time. This paper is intended to fill this gap by using plain language and concrete examples to elucidate the *p*-value confusion from its root, to intuitively describe the true meaning of *p*-values, to illuminate the consequences of the confusion, and to present a viable alternative to the conventional *p*-value.

## Main text

### The root of confusion

The p-value confusion began 100 years ago when the father of modern statistics, Ronald Aylmer Fisher, formed the paradigm of significance testing. But it should be noted Fisher bears no blame for the misconception; it is the users who tend to muddle Fisher’s significance testing with hypothesis testing developed by Jerzy Neyman and Egon Pearson. To clarify the confusion, this section uses concrete examples and plain language to illustrate the essence of significance and hypothesis testing and to explicate the difference between the *p*-value and the type I error.

#### Significance testing

Suppose a painkiller has a proven track record of lasting for 24 h and now another drug manufacturer claims its new over-the-counter painkiller lasts longer. An investigator wants to test if the claim is true. Instead of collecting data from all the patients who took the new medication, which is often infeasible, the investigator decided to randomly survey 50 patients to gather data on how long (hours) the new painkiller lasts. Thus, the investigator now has a random variable $\overline{X}$, the average hours from a sample of 50 patients. This is a random variable because the 50 patients are randomly selected, and nobody knows what value this variable will take before conducting the survey and calculating the average. Nevertheless, each survey does produce a fixed number, $\overline{x}$, which itself is not a random variable, rather it is a realization or observation of the random variable $\overline{X}$ (hereafter, let $\overline{X}$ denote a random variable and $\overline{x}$ denote a fixed value, an observation of $\overline{X}$).

Intuitively, if the survey yielded a value (average hours the painkiller lasts) very close to 24, say, 23 or 25, the investigator would not believe the new painkiller is worse or better. If the survey came to an average of 32 h the investigator would believe it indeed lasts longer. However, it would be hard to form an opinion if the survey showed an average of 22 or 26 h. Does the new painkiller really last shorter, longer, or it is due to random chance (after all, only 50 patients were surveyed)?

This is where the significance test formulated by Fisher in the 1920s comes in. Note that although modern significance testing began with the Student’s *t*-test in 1908, it was Fisher who extended the test to the testing of two samples, regression coefficients, as well as analysis of variance, and created the paradigm of significance testing.

In Fisher’s significance testing, the Central Limit Theorem (CLT) plays a vital role, which states that given a population with a mean of μ and a variance of σ^2^, regardless of the shape of its distribution, the sample mean statistic $\overline{X}$ has a normal distribution with the same mean μ and variance σ^2^/n, or $\frac{\left(\overline{X}-\upmu \right)}{\sigma /\sqrt{n}}$ has a standard normal distribution with a mean of 0 and a variance of 1, as long as the sample size *n* is large enough. In practice, the distribution of the study population is often unknown and *n* ≥ 30 is considered sufficient for the sample mean statistic to have an approximately normal distribution.

In conducting the significance test, a null hypothesis is first formed, i.e., there is no difference between the new and old painkillers, or the new painkiller also lasts for 24 h (the mean of $\overline{X}$ = μ =24). Under this assumption and based on CLT, $\overline{X}$ is normally distributed with a mean of 24 and a variance of σ^2^/50. Assume σ^2^ = 200 (typically σ^2^ is unknown but can be estimated), then $\overline{X}$ has a normal distribution *N (24, 2)*, or $Z=\left(\overline{X}-24\right)/2$ has a standard normal distribution with a mean of 0 and standard deviation of 1 (Z is a standardized random variable). The next step is to calculate z = $\mid \overline{x}-24\mid /2$ based on the survey data and then find the *p*-value or the probability of |Z| > z from a normal distribution table (z is a fixed value or an observation of Z). Fisher suggested if the *p*-value is smaller than 0.05 then the hypothesis is rejected. He argued that the farther the sample mean $\overline{x}$ from the population mean μ, the smaller the *p*-value, the less likely the null hypothesis is true. Just as Fisher stated, “Either an exceptionally rare chance has occurred or the theory [H_0_] is not true [11].”

Based on this paradigm, if the survey came back with an average of 26 h, i.e., $\overline{x}=26,$ then *z* = 1 and *p* = 0.3173, as a result, the investigator accepts the null hypothesis (orthodoxically, fails to reject the null hypothesis), i.e., the new painkiller does not last longer and the difference between 24 and 26 h is due to chance or random factors. On the other hand, if the survey revealed an average of 28 h, i.e., $\overline{x}=28,$ then *z* = 2, and *p* = 0.0455, thus the null hypothesis is rejected. In other words, the new painkiller is deemed lasting longer.

Now, can the *p*-value, 0.0455, be interpreted as the probability of the type I error, or only 4.55% chance the new painkiller does not last longer (no difference), or the probability that the difference between 24 and 28 h is due to chance, or the investigator could make a mistake by rejecting the null hypothesis but only wrong about 4.55% of the time? The answer is No.

So, what is a *p*-value? Precisely, a p-value tells us how often we would see a difference as extreme as or more extreme than what is observed *if there really were no difference*. Drawing a bell curve with the *p*-value on it will readily delineate this definition or concept.

In the example above, *if the new painkiller also lasts for 24 h,* the p-value of 0.0455 means there is a 4.55% chance that the investigator would observe $\overline{x}\le 20$ or $\overline{x}\ge 28$; it is not 4.55% chance the new painkiller also lasts for 24 h. It is categorically wrong to believe the *p*-value is the probability of the null hypothesis being true (there is no difference), or *1 – p* is the probability of the null hypothesis being false (there is a difference) because the *p*-value is deduced based on the premise that the null hypothesis is true. The p-value, a conditional probability given H_0_ is true, is totally invalidated if the null hypothesis is deemed not true.

In addition, *p*-values are data-dependent: each test (survey) produces a different p-value; for the same analysis, it is illogical to say the error rate or the type I error is 31.73% based on one sample (survey) and 4.55% based on another. There is no theoretical or empirical basis for such frequency interpretations. In fact, Fisher himself was fully aware that his *p*-value, a relative measure of evidence against the null hypothesis, does not bear any interpretation of the long-term error rate. When the *p*-value was misinterpreted, he protested the p-value was not the type I error rate, had no long-run frequentist characteristics, and should not be explained as a frequency of error if the test was repeated [12].

Interestingly, Fisher was an abstract thinker at the highest level, but often developed solutions and tests without solid theoretical foundation. He was an obstinate proponent of inductive inference, i.e., reasoning from specific to general, or from sample to population, which is reflected by his significance testing.

#### Hypothesis testing

On the contrary, mathematicians Jerzy Neyman and Egon Pearson dismissed the idea of inductive inference all together and insisted reasoning should be deductive, i.e., from general to specific. In 1928, they published the landmark paper on the theoretical foundation for a statistical inference method that they called “hypothesis test [12].” In the paper, they introduced the concepts of alternative hypothesis H_1,_ type I and type II errors, which were groundbreaking. The Neyman and Pearson’s hypothesis test is deductive in nature, i.e., reasoning from general to particular. The type I and type II errors, which must be set ahead, formulate a “rule of behavior” such that “in the long run of experience, we shall not be too often wrong,” as stated by Neyman and Pearson [13].

The hypothesis test can be illustrated by a four-step process with the painkiller example.

The first step is to lay out what the investigator seeks to test, i.e. to establish a null hypothesis, H_0,_ and an alternative hypothesis, H_1_:

$$ {\mathrm{H}}_0:\mathrm{mean}\ \mathrm{of}\kern0.75em \overline{X}=\upmu =24 $$

$$ {\mathrm{H}}_1:\mathrm{mean}\ \mathrm{of}\ \overline{X}=\upmu =28 $$

The second step is to set the criteria for the decision, or to specify an acceptable rate of mistake if the test is conducted many times. Specifically, that is to set the probability of the type I error, α, and the probability of the type II error, β.

A type I error refers to the mistake of rejecting the null hypothesis when it is true (claiming the treatment works or the new drug lasts longer but actually it does not). Conventionally and almost universally, the probability of the type I error or α is set to 0.05, which means 5% of the time one will be wrong if carrying out the test many times. A type II error is the failure to reject the null hypothesis that is not true; the probability of the type II error, β, is conventionally set as 0.2, which is equivalent to a power of 0.8, the probability of detecting the difference if it exists. Table 1 summarizes the type I and type II errors.

**Table 1:** Type I and Type II Errors

	Null hypothesis (H0) is true	Null hypothesis (H0) is false
Reject the null hypothesis	Type I error False positive	Correct decision True positive
Accept the null hypothesis	Correct decision True negative	Type II error False negative

**Fig. 1:**
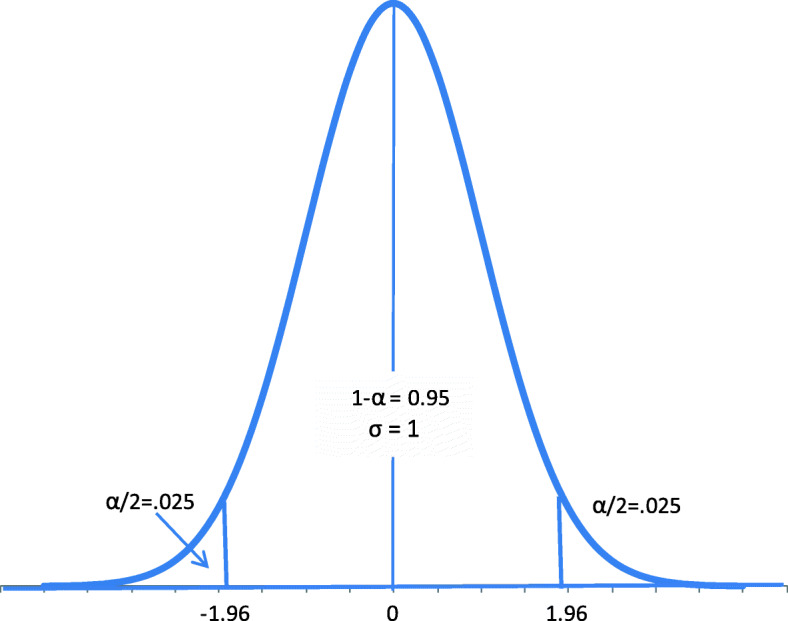
Standard Normal Distribution with Critical Value 1.96

The third step is to select a statistic and the associated distribution for the test. For the painkiller example, the statistic is Z = ($\overline{X}-24$)/2, and the distribution is the standard normal. Because the type I error has been set to 0.05 and *Z* has a standard normal distribution under the null hypothesis, as shown in Fig. 1, 1.96 becomes the critical value, − 1.96 *≤ z ≤* 1.96 becomes the acceptance region, and *z < − 1.96* or *z > 1.96* becomes the rejection regions.

The final step is to calculate the z value and make a decision. Similar to significance testing, if the survey resulted in $\overline{x}=26,$ then z = 1 < 1.96 and the investigator accepts the null hypothesis; if the survey revealed $\overline{x}=28,$ then z = 2 > 1.96 and the investigator rejects the null hypothesis and accepts the alternative hypothesis. It is interesting to note, in significance testing, “one can never accept the null hypothesis, only failed to reject it,” while that is not the case in hypothesis testing.

Unlike Fisher’s significance test, the hypothesis test possesses a nice frequency explanation: one can be wrong by rejecting the null hypothesis but cannot be wrong more than 5% of the time in the long run if the test is performed many times. Quite intuitively, every time the null hypothesis is rejected (when *z < − 1.96* or *z > 1.*96) there is a chance that the null hypothesis is true, and a mistake is made. When the null hypothesis is true, Z = ($\overline{X}-24$)/2 is a random variable with a standard normal distribution as shown in Fig. 1, thus 5% of the time z = ($\overline{x}-24$)/2 would fall outside (− 1.96, 1.96) and the decision will be wrong 5% of the time. Of course, when the null hypothesis is not true, rejecting it is not a mistake.

Noticeably, the *p*-value plays no role in hypothesis testing under the framework of the Neyman-Pearson paradigm [12, 14]. However, most, including many statisticians, are unaware that *p*-values and significance testing created by Fisher are incomparable to the hypothesis testing paradigm created by Neyman and Pearson [14, 15], and many statistics textbooks tend to cobble them together [2, 14]. The near-universal confusion is, at least in part, caused by the subtle similarities and differences between the two tests:

1. Both the significance and hypothesis tests use the same statistic and distribution, for example, Z = ($\overline{X}-24$)/2 and *N* (0, 1).
2. The hypothesis test compares the observed *z* with the critical value 1.96, while the significance test compares the *p*-value (based on z) to 0.05, which are linked by *P*(|*Z*| > 1.96) = 0.05.
3. The hypothesis test sets the type I error α at 0.05, while the significance test also uses 0.05 as the significance level.

One of the key differences is, for the *p*-value to be meaningful in significance testing, the null hypothesis must be true, while this is not the case for the critical value in hypothesis testing. Although the critical value is derived from α based on the null hypothesis, rejecting the null hypothesis is not a mistake when it is not true; when it is true, there is a 5% chance that z = ($\overline{x}-24$)/2 will fall outside (− 1.96, 1.96), and the investigator will be wrong 5% of the time (bear in mind, the null hypothesis is either true or false when a decision is made). In addition, the type I error and the resultant critical value is set ahead and fixed, while the *p*-value is a moving “target” varying from sample to sample even for the same test.

As if it was not confusing enough, the understanding and interpretation of *p*-values are also complicated by non-experimental studies where model misspecifications and even p-hacking are common, which often misleads the audience to believe the model and the findings are valid for its small *p*-values [16]. In fact, p-values have little value in assessing if the relationship between an outcome and exposure(s) is causal or just an artifact of confounding – one cannot claim the use of smartphones causes gun violence even if the *p*-value for their correlation is close to zero. To see the p-value problem at its core and to elucidate the confusion, the discussion of p-values should be in the context of experimental designs such as randomized controlled trials where the model or the outcome and exposure(s) are correctly specified.

### The Link between *P*-values and Type I Errors

The p-value fallacy can be readily quantified under a Bayesian framework [7, 17, 18]. However, “those ominous words [Bayes theorem], with their associations of hazy prior probabilities and abstruse mathematical formulas, strike fear into the hearts of most us, clinician, researcher, and editor alike [19],” as Frank Davidoff, former Editor of the *Annals of Internal Medicine*, wrote. It is understandable but still unfortunate that Bayesian methods such as Bayes factors, despite their merit, are still considered exotic by the medical research community.

Thanks to Sellke, Bayarri, and Berger, the difference between the *p*-value and the type I error is quantified [7]. Based on the conditional frequentist approach, which was formalized by Kiefer and further developed by others [20–23], Berger and colleagues established the lower bound of the error rate P(H_0_│| Z| >z_0_) or the type I error given the p-value [7]: After converting to PDF, the left side of the equation below shows a mail sign rather than

$$ \mathrm{P}\left({\mathrm{H}}_0\vert |\mathrm{Z}|>{\mathrm{z}}_0\right)=\upalpha \left(\mathrm{p}\right)={\left\{1+{\left[-\mathrm{e}\times \mathrm{p}\times \ln \left(\mathrm{p}\right)\right]}^{-1}\right\}}^{-1}. $$

As shown, the lower bound equation is mathematically straightforward. Noteworthy is that the derivation of the lower bound is also ingeniously elegant (a simplified proof is provided in the Supplementary File for those who are interested in it). The relationship between *p*-values and type I errors (lower bound) can be readily seen from Table 2 showing some of the commonly reported results [7].

**Table 2:** *P*-values and Associated Type I Error Probabilities (lower bound)

*P*-value	0.20	0.15	0.10	0.05	0.02	0.01	0.005	0.001
α(p)	0.465	0.436	0.385	0.289	0.175	0.111	0.067	0.018

As seen in Table 2, the difference between *p*-values and the error probabilities (lower bound) is quite striking. A p-value of 0.05, commonly misinterpreted as only 5% chance the treatment does not work, seems to offer strong evidence against the null hypothesis; however, the true probability of the treatment not working is at least 0.289. Keep in mind, the relationship between the p-value and the type I error is the lower bound; in fact, many prefer to report the upper bound [6, 7].

The discrepancy between the p-value and the lower-bound error rate explains the big puzzle of why so many wonder drugs and treatments worldwide lose their amazing power outside clinical trials [24–26]. This discrepancy likely also contributes to the frequently reported contradictory findings on risk factors and health outcomes in observational studies. For example, an early study published in the *New England Journal of Medicine* found drinking coffee was associated with a high risk of pancreatic cancer [27]. The finding became a big headline in *The New York Times* [28] and the leading author and probably many frightened readers stopped drinking coffee. Later studies, however, concluded the finding was a fluke [29, 30]. Likewise, the *p*-value fallacy may also contribute to the ongoing confusion of dietary fat intake and heart disease. On the one hand, a meta-analysis published in *Annals of Internal Medicine* in 2014 concluded “Current evidence does not clearly support cardiovascular guidelines that encourage high consumption of polyunsaturated fatty acids and low consumption of total saturated fats [31].” On the other hand, in the 2017 recommendation, the American Heart Association (AHA) stated “Taking into consideration the totality of the scientific evidence, satisfying rigorous criteria for causality, we conclude strongly that lowering intake of saturated fat and replacing it with unsaturated fats, especially polyunsaturated fats, will lower the incidence of CVD [32].”

In short, the misunderstanding and misinterpretation of the relationship between the *p*-value and the type I error all too often exaggerate the true effects of treatments and risk factors, which in turn leads to conflicting findings with real public health consequences.

### The future of *P*-values

It is readily apparent that the p-value conundrum poses a serious challenge to researchers and practitioners alike in healthcare with real-life consequences. To address the p-value complications, some believe the use of *p*-values should be banned or discouraged [33, 34]. In fact, since 2015, *Basic and Applied Social Psychology* has officially banned significance tests and p-values [35], and *Epidemiology* has a longstanding policy discouraging the use of significance testing and *p*-values [36, 37]. On the other hand, many are against a total ban [38, 39]. *P*-values do possess practical utility -- they offer insight into what is observed and are the first line of defense against being fooled by randomness. You would be more suspicious of a coin being fair if nine heads turned up after ten flips versus, for example, if seven heads did. Similarly, you would like to see how strong the evidence is against the null hypothesis: say, a p-value of 0.0499 or 0.0001.

“It is hopeless to expect users to change their reliance on *p*-values unless they are offered an alternative way of judging the reliability of their conclusions [40].” Rather than banning the use of p-values, many believe the conventional significance level of 0.05 should be lowered for better research reproducibility [41]. In 2018, 72 statisticians and scientists made the case for changing *p* < 0.05 to *p* < 0.005 [42]. Inevitably, like most medical treatments, the proposed change is accompanied by some side effects: For instance, to achieve the same power of 80%, α = 0.005 requires a 70% larger sample size compared to α = 0.05, which could lead to fewer studies due to limited resources [43].

Other alternatives (e.g., second-generation *p*-values [44], and analysis of credibility [45]) have been proposed in the special issue of the *American Statistician*; however, no consensus was reached. As a result, instead of recommending a ban of *p*-values, the accompanying editorial of the special issue called for an end of statistical significance testing [46]: “‘statistically significant’ – don’t say it and don’t use it [10].”

Will researchers and medical journals heed the “mandate” banning significance testing? It does not seem to be likely, at least thus far. Even if they do, it is no more than just a quibble – a significance test is done as long as the *p*-value is produced or reported – anyone seeing the result would know the p-value is greater or less than 0.05; the only difference is “Don’t ask, don’t tell.”

In any event, it is the right call to end dichotomizing the p-value and using it as the sole criterion to judge the results [47]. There is no practical difference between *p* = 0.049 and *p* = 0.051, and “God loves the .06 nearly as much as the .05 [48].” Furthermore, not all the results with a *p*-value close to 0.05 are valueless. Doctors and patients need to put *p*-values into context when making treatment choices, which can be well illustrated by a hypothetical but not unrealistic example. Suppose a study finds a new procedure (a kind of spine surgery) is effective in relieving debilitating neck and back pain with a *p*-value of 0.05, but when the procedure fails, it cripples the patient. If the patient believes there is only a 5% chance the procedure does not work or fails, he or she would probably take the chance. However, after learning the actual chance of failure is nearly 30% or higher based on the calibrated p-value, one would probably think twice. On the other hand, even if the p-value is 0.1 and the real chance of failure is nearly 40% or higher, if it does not cause serious side effects when the procedure fails, one would probably like to give it a try.

Taken together, in medicine or healthcare, the use of *p*-values needs more context (the balance of harms and benefits) than thresholds. However, banning significance testing and accepting uncertainty as called for by the editorial of the special issue are not enough [10]. When making treatment decisions, what researchers, practitioners, and patients alike need to know is the probability that a treatment does or does not work (the type I error). In this regard, the calibrated *p*-value, compared to other proposals [44, 45], offers several advantages: (1) It provides a lower-bound, (2) it is fully frequentist although it can have a Bayesian interpretation, (3) it is easy to understand, and (4) it is easy to implement. Of course, other recommendations for improving the use of *p*-values may work well under different circumstances such as improving research reproducibility [49, 50].

## Conclusions

In medical research and practice, the p-value produced from significance testing has been widely misconstrued as the probability of the type I error, or the probability a treatment does not work. This misunderstanding comes with serious consequences: poor research reproducibility and inflated medical treatment effects. For a *p*-value of 0.05, the type I error or the chance a treatment does not work is not 5%; rather, it is at least 28.9%. Nevertheless, banning significance testing and accepting uncertainty, albeit well justified in many circumstances, offer little to apprise clinicians and patients of the probability a treatment will or will not work. In this respect, the calibrated p-value, a link between the p-value and the type I error, is practical and instructive.

In short, a long-overdue effort to understand *p*-values correctly is urgently needed and better education on statistical reasoning including Bayesian methods is desired [15]. Meanwhile, a rational action that medical journals can take is to require authors to report both conventional p-values and calibrated ones in research papers.

## Supplementary information

**Additional file 1.**

## Data Availability

Not applicable.

## Abbreviations

FDA: U.S. Food & Drug Administration
JAMA: Journal of the American Medical Association
ASA: American Statistical Association
CLT: Central Limit Theorem
AHA: American Heart Association
