# Supplementary material for: P-values – a chronic conundrum
Source: BMC Med Res Methodol. 2020 Jun 24;20:167. doi: 10.1186/s12874-020-01051-6 (PMC7315482; doi:10.1186/s12874-020-01051-6)
Supplement: Supplementary file 1 — Additional file 1. [file 12874_2020_1051_MOESM1_ESM.pdf]

# P-Values – A Chronic Conundrum

Derivation of the Calibrated P-values

The lower bound is derived based on conditional frequentist testing (Kiefer, 1977 JASA; Berger, Brown and Wolpert, 1994 AOS). After assessing different options, Berger and colleagues found p-value conditioning and the Beta distribution $Beta(\xi, 1) = \xi p^{\xi-1}$ for H_1 most desirable for practical purposes given that p has a uniform distribution U(0, 1) under H_0. With the conditioning statistic $C = \max\{p_0, p_1\} = \max\{p, \xi p^{\xi-1}\}$, where p_0 is the p-value from testing H_0 against H_1, and p_1 is the p-value from testing H_1 against H_0, the resultant conditional frequentist test is devised as (Sellke, Bayarri and Berger, 2001 Am Stat):

If $p \leq \eta$, reject H_0 and report type I conditional error probability $\alpha_\xi(p) = (1 + \xi p^{\xi-1})^{-1}$; and on the other hand, if $p > \eta$, accept H_0 and report type II conditional error probability $\beta_\xi(p) = (1 + \xi^{-1} p^{1-\xi})^{-1}$, where η is the solution of the equation $\eta = 1 - \eta^{\xi}$.

It can be readily shown that the lower bound of $\alpha_\xi(p) = (1 + \xi p^{\xi-1})^{-1}$ is $\alpha(\mathrm{p}) = \{1 + [-\mathrm{e} \times \mathrm{p} \times \ln(\mathrm{p})]^{-1}\}^{-1}$; in other words, $\alpha_\xi(p) = (1 + \xi p^{\xi-1})^{-1} \geq \alpha(\mathrm{p}) = \{1 + [-\mathrm{e} \times \mathrm{p} \times \ln(\mathrm{p})]^{-1}\}^{-1}$, for $p < e^{-1}$.

It is worth emphasizing that the lower bound holds for any value of $0 < \xi \leq 1$, and the calibration is fully frequentist although it can have a Bayesian interpretation. Further, although the class of $Beta(\xi, 1)$ densities of p under H_1 may not be "general" enough to cover all the possible alternatives theoretically, the lower bound indeed meets practical needs since it is also valid over a variety of nonparametric alternatives (Sellke, Bayarri and Berger, 2001 Am Stat).
